# Vitamin D Food Fortification Strategies on Population-Based Dietary Intake Data Using Mixed-Integer Programming

**DOI:** 10.3390/foods12040698

**Published:** 2023-02-06

**Authors:** Sayantan Sengupta, Tue Christensen, Gitte Ravn-Haren, Rikke Andersen

**Affiliations:** 1 Research Group for Risk Benefit, National Food Institute, Technical University of Demark, 2800 Kongens Lyngby, Denmark; 2 Research Group for Nutrition, Sustainability and Health Promotion, National Food Institute, Technical University of Demark, 2800 Kongens Lyngby, Denmark

**Keywords:** fortification, vitamin D, food, dietary intake data, integer programming, optimization

## Abstract

The dietary vitamin D intake of the Danish population is low, and food fortification is a strategy to increase intake. This paper explores the possibility of vitamin D fortification on the current population food intake in Denmark, such that the population receives adequate amounts of vitamin D without having to change current dietary patterns. A mixed-integer programming approach is used to arrive at a solution for the optimal fortification required at each food group level so that the majority of the population receive the minimum intake of average requirement (AR) and do not exceed the tolerable upper intake level (UL). The method shows a significant increase in vitamin D intake compared to the current scenario, keeping a neutral approach towards preferences of one food group over others. The method can also be fine-tuned in different scenarios where certain food group preferences are known, which can be encoded into the model in the form of constraints.

## 1. Introduction

Vitamin D deficiency has an effect on bone health and is also linked to nonskeletal diseases and increased mortality [1,2]. Vitamin D status (measured as plasma 25-hydroxyvitamin D (25OHD)) can be improved by increasing sun exposure, supplement intake, and/or dietary intake.

Sun exposure is limited in areas such as the Nordic countries, where the UVB radiation is too low for sufficient cutaneous vitamin D production during wintertime [3]. Additionally, increased sun exposure is a problematic strategy to increase vitamin D status due to the risk of skin cancer [4].

Randomized controlled trials have documented that the increased intake of food supplements is an effective strategy to increase plasma 25OHD [1]. Advising food supplement use can be a useful strategy when targeted at population groups during specific life cycle stages, such as for the elderly at nursing homes [5]. As a strategy to increase the vitamin D intake in the general population, the intake of food supplements is a less useful strategy since regular life-long food supplement intake is challenging to remember for most people, and it requires repetitive public campaigns. Furthermore, food supplement intake is skewed across age and sociodemographic groups [6]. The use of high-dose food supplements increases the risk of exceeding the tolerable upper intake level (UL) [7].

There are only a few good natural dietary sources of vitamin D. The main food groups contributing to vitamin D intake are typically fish, meat, milk, cheese, and eggs. The habitual dietary intake of vitamin D is lower than the recommended intake [8,9]. 

Food fortification is therefore used in some countries as a strategy to increase dietary intake. For example, Finland’s vitamin D fortification policy has been very successful since 2003, when the systematic voluntary fortification of fluid milk products and fat spreads was initiated. The vitamin D status of Finnish adults has improved, and the increase is mainly explained by the fortification of fluid milk and vitamin D food supplement use [10]. Even though the Finnish fortification policy is voluntary, almost all Finnish manufacturers follow it [9]. In 2018, the mandatory fortification policy in Sweden was expanded to include all milk with a fat content <3% and sour milk products, lactose-free products, vegetable-based alternatives, and fluid margarines [11]. Butter, margarine, and some low-fat milk products are fortified voluntarily in Norway [9].

Even though the fortification of all margarine products was mandatory in Denmark from the mid-1930s up until mid-1985 (when it was banned) [12], Denmark does not have a long tradition of fortifying foods. Presently, vitamin D food fortification needs to be approved by the Danish Veterinary and Food Administration. The approval is based on an individual case-by-case risk assessment. Generally, the addition of vitamin D to certain products is accepted by the authorities, but few vitamin D-fortified products are on the Danish market [9]. The efficiency of a food fortification program depends on which foods are fortified. The vitamin D fortification of several different food groups has been suggested as optimal [13]. 

The main aim of this paper is to explore the possibility of vitamin D fortification in the current population of Denmark’s food intake, such that the population receives an adequate amount of vitamin D without having to change current dietary patterns. A mixed-integer programming approach is used to arrive at a solution for the optimal fortification required at each food group level so that the majority of the population’s intake is above the average requirement (AR) [2] and does not exceed the tolerable upper intake level (UL) [7]. The quantitative values of the AR, RI, and UL are shown in Table 1.

## 2. Materials and Methods

Dietary intake data from the Danish national survey of Dietary Habits and Physical Activity 2011–2013 [8] are used for this analysis. The survey is based on 7-day dietary recordings, recipe collections, and food composition data [14]. Data from registration is interpreted into food items from the food composition database using the recipe collection. Each food item belongs to a food group (‘Milk and milk products’, ‘Cheese and cheese products’, ‘Ice cream, fruit ice, and other edible ices’, ‘Cereals and cereal products’, ‘Vegetables and vegetable products’, ‘Fruit and fruit products’, ‘Meat and meat products’, ‘Fish and fish products’, ‘Poultry and poultry products’, ‘Eggs and egg products’, ‘Fats, oils, and their products’, ‘Sugar, honey and products thereof’, ‘Beverages’, ‘Spices and other ingredients’, ‘Other foods’, ‘Potato and products thereof’, and ‘Juice’) (Table 2).

Mixed-integer programming is a variation of the linear programming method. Linear programming solves a linear set of equations with some linear constraints and the variables being solved can take any continuous values with respect to the constraints. Integer programming, on the other hand, also solves for linear equations with linear constraints, with the exception that the variables can only take integer values (whole numbers), not fractions or decimals. Mixed-integer programming includes these two aspects to achieve greater complexity. In simple linear programming formulation, the solver does not have the flexibility to decide which variables (food groups) to choose from for fortification. 

In many of these cases, the easiest solution is to choose one food group and find the perfect fortification level that can satisfy the chosen constraints. Mixed-integer programming, on the other hand, provides more choices for the selection of food groups in terms of constraints. 

The first step is to create a food composition matrix. This matrix is created from the dietary intake data and consists of food groups, the average intake of the population (in g/day), and the vitamin D concentration of each food group (µg/g), as shown in Table 3. The vitamin D concentration for each food group is derived by aggregating over all food items in each group. All the fortifiable food items can be categorized into a few food groups, as shown in Table 2. Henceforth, all the food groups are referred to by their food group number from Table 2. Although vitamins D2 and D3 do not have the same potency for increasing 250HD levels, we assume that vitamin D3 is always used as it is contained in the majority of food items.

As the consumption is constant, the vitamin D concentration becomes the variable in this formulation which, when solved for, provides the optimal solution for fortification. The formulation is as follows:

(1) $$maximize\;:\sum^{\;}food\;group\;intake_{i}*new\;vitamin\;D\;concentration_{i}\;$$

(2) $$such\;that:\;\sum^{\;}food\;group\;intake_{i}*new\;vitamin\;D\;concentration_{i}>AR$$

(3) $$such\;that:\;\sum^{\;}food\;group\;intake_{i}*new\;vitamin\;D\;concentration_{i}=RI$$

(4) $$such\;that:\;\sum^{\;}food\;group\;intake_{i}*new\;vitamin\;D\;concentration_{i}<UL$$

(5) $$such\;that:\;\sum^{\;}new\;vitamin\;D\;concentration_{i}>food\;chosen_{i}\times 0.1$$

(6) $$such\;that:\;\sum^{\;}new\;vitamin\;D\;concentration_{i}<food\;chosen_{i}*10000$$

(7) $$such\;that:\;\sum^{\;}new\;vitamin\;D\;concentration_{i}<current\;fortification+constant\;1\;\;\;\;\;$$

(8) $$such\;that:\;\sum^{\;}food\;chosen_{i}<Constant\;2$$

The above formulation has an objective function that represents the maximization of the total vitamin D intake of the population across all groups. There are two variables that are being solved for: the continuous variable $new\;vitamin\;D\;concentration_{i}$ and the integer variable (which takes values of either 0 or 1) $food\;chosen_{i}$. The integer variable also acts as an indicator variable and can help to choose/prefer one food group over the other. This general framework of combining linear programming with integer constraints can be fine-tuned by practitioners based on specific situations and domain knowledge.

Equation (1) is the objective function that formulates total vitamin D intake and must be maximized. Equations (2)–(4) represent the vitamin D constraints for the average requirement (AR), recommended intake (RI), and tolerable upper intake level (UL). Equations (5) and (6) combine the continuous variable and the integer variable into the formulation. These two equations effectively mean that we are giving $new\;vitamin\;D\;concentration_{i}$ importance only if $food\;chosen_{i}$ is 1 (chosen). This way, we avoid direct multiplication and maintain a linear problem structure. Equation (7) has a special purpose of keeping the new values of vitamin D fortification close to the current values of fortification. The *constant* 1 is kept at a low value (0.2 for the whole population, 1 for age group 4–10 years, and 0.1 for age group 11–17 years) to not let the new values of fortification deviate too much from the current level. The value of this constant arrives after fine tuning. Equation (8) is optional, where certain food groups can be preferred to be chosen over others if the situation demands, which is implemented in an ‘*either–or’* logic. For example, if we *either* prefer food group 1 *or* food group 2 to be chosen in the final solution for fortification, then the constraint looks like $food\;chosen_{1}+food\;chosen_{2}<1$. This ensures that the sum of these two binary variables is at most 1, which means only one of them can be included in the optimal solution but not both. In this analysis, we exclude the constraint of keeping a neutral preference for all the food groups.

Four models are trained for four different age groups. Each model has the same equations and constraints as mentioned above, but differs depending on which population subgroup it has been applied to. As different subpopulations have different initial vitamin D exposures, the model parameters are tuned specifically for those subgroups: Model 1 for the entire population (age group 4–75 years), Model 2 for the age group 11–17 years, Model 3 for the age group 18–69 years, and Model 4 for the age group 4–10 years. The above formulation is solved in Python (3.9 with Anaconda installation) with the optimization package Pulp (2.60). The python code can be downloaded at: https://gitlab.gbar.dtu.dk/says/Vitamin_D_fortification_optimization_model.git (accessed on 4 January 2023).

## 3. Results and Discussion

The results of all the models are shown in Table 4. Four food groups are chosen by Model 1 and 3, whereas six food groups are chosen by Model 2 and two food groups by Model 4 for fortification. Food group 3 is the only group that appears across all the three models as an important food group for fortification. One interesting observation from the results is that the new fortification level suggested by Model 1 and Model 2 is clipped around 0.2, Model 3 around 0.1, and Model 4 around 1. This is due to the effect of Equation (7), which constrains the new fortification to be not more than the current fortification by constant 1. This constraint also plays a significant role in distributing the overall gap between the current fortification and the optimal fortification across more food groups, rather than selecting one food group and an exceptionally high fortification of that. A simple linear optimization formulation is more likely to not distribute across more food groups. One disadvantage, however, is the economic cost associated with fortifying across multiple food groups, which can be reduced if only one food group is chosen.

**Figure 1:**
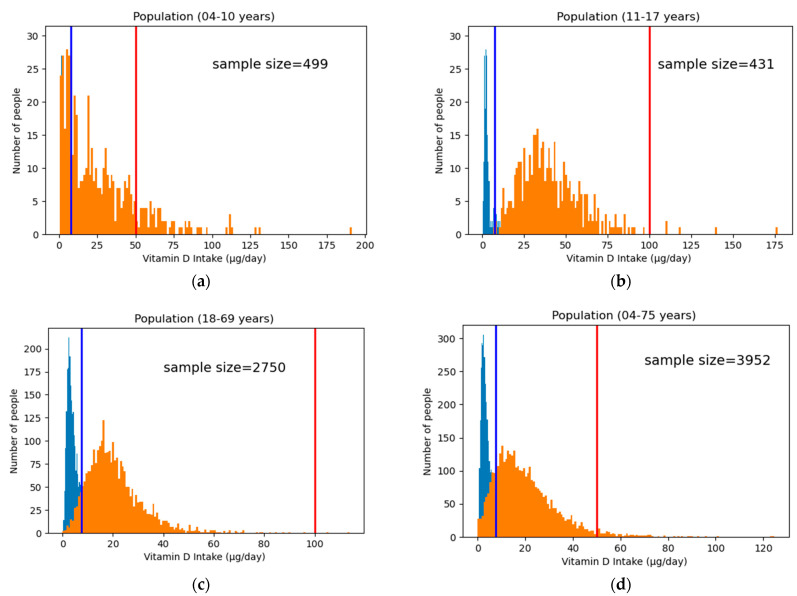
Population distribution of vitamin D intake of the four models trained on their respective age groups. The blue histogram represents the current vitamin D intake, whereas the orange histogram represents the new intake after fortification. The blue vertical line shows the AR, and the red line shows the UL: (**a**) intake distribution of Model 4 trained on the age group 4–10 years; (**b**) intake distribution of Model 2 trained on the age group 11–17 years; (**c**) intake distribution of Model 3 trained on the age group 18–69 years; and (**d**) intake distribution of Model 1 trained on the entire population age group 4–75 years.

To compare the effect of the new fortification on their respective population age groups, the new fortification values must be dispersed from the food group level to the individual food items. In this analysis, the new fortification values are substituted for each food item in that food group. This assumption does not consider the difference in the consumption pattern of each food item in a food group and thus gives equal weight in assigning the fortification values. This necessary step helps to derive the new intake distribution of vitamin D in the population, and the results are shown in Table 5 and Figure 1.

There is a remarkable decrease in the population whose estimated daily vitamin D intake is below the AR in all four age groups. In the total population (age group 4–75), approximately 83% were below the AR in the current scenario (no food fortification), which falls to 14% with the new fortification strategy. Similarly, the percentage of the population below RI falls from 90% to only 23% and the mean intake increases from roughly 5 µg/day to 20 µg/day. Similar improvements can be observed across all age groups, which shows that all the four optimization models are successful in increasing the vitamin D intake in the population, keeping current food consumption steady. One important aspect to be noted in the modeling is the choice of UL. From Table 1, the UL is 50 µg/day for the age group 4–10, whereas it is 100 µg/day for the rest of the population. The 4–10 age group becomes the limiting factor while modeling the entire population (4–75 years), and thus, the UL is kept at 50 µg/day for Model 1 (Figure 1d). The methodology presented so far provides a blueprint to design, execute, and model certain age groups of the specific population, taking into consideration the fine-tuning of various parameters.

**Figure 2:**
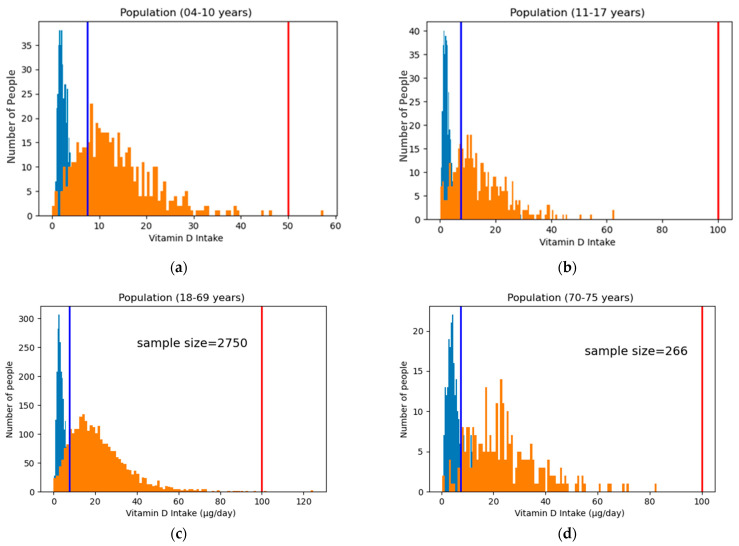
Individual age group distribution of vitamin D extracted from Model 1. These graphs show the effect of changes in each age range due to Model 1, which is trained on the entire population. The blue histogram represents the current vitamin D intake, whereas the orange histogram represents the new intake after fortification. The blue vertical line shows the AR, and the red line shows the UL: (**a**) intake distribution for the age group 4–10 years); (**b**) intake distribution in the age group 11–17 years; (**c**) intake distribution in the age range of 18–69 years; (**d**) and intake distribution in the age range of 70–75 years.

To realize and implement this method for practical use, only the model trained on the entire population should be considered. Fortification strategies are usually devised for the entire population as any age-specific targeted food groups are not taken into consideration. The next step would be to evaluate how a model trained on the entire population performs, which is Model 1. Figure 2 shows the vitamin D intake in both scenarios (before and after fortification) for different age groups for Model 1. First, we check the sensitive age group (4–10 years) not exceeding the UL. It can be seen from Figure 2 that the number of people exceeding the UL is 1 out of 499 in the age group 4–10 years, 3 out of 2750 in the age group 18–69 years, and 0 in the age groups 11–17 years and 70–75 years. This shows the robustness of Model 1 with respect to adhering to the different ULs for different age groups. Strict adherence to the UL makes it safe for the population. Table 6 presents a detailed summary of the effect of Model 1 on different age groups. There is a significant increase in the percentage of the population above the AR after fortification for all age groups.

However, there are a few limitations and aspects of the model that require a more in-depth analysis. The first is the choice of food groups. From Table 3, many of the food groups considered for modeling have zero vitamin D concentration in the current scenario. If we discard those food groups and build an optimization model only on food groups with nonzero vitamin D concentration, then the population distribution after fortification appears differently. This is a choice to be made by the practitioner. In this paper, all the food groups, irrespective of their current vitamin D level, are taken into the model such that they can be fortified. Second, the way of assigning new fortification values of a food group to all its food items will also affect the shape of the population distribution. In this paper, the optimal fortification estimates obtained from Model 1 for all four food groups are assumed as the empirical mean for the food items in their respective food groups. It is equally true that not all the food items are consumed in equal proportions within a food group. The weighted means is an alternate way of assigning these values from food group level to the individual food items. Third, the population distribution of vitamin D intake after fortification is underestimated in the practical scenario. This is because only four food groups are chosen for fortification, and a suitable fortification estimate is found, correspondingly, in Model 1. The food groups that are not chosen are assigned zero vitamin D. However, this will not be true in the practical scenario as the rest of the food groups that are not chosen by the model will still retain the current values of the vitamin D. Due to this, the estimation of vitamin D in the population is underestimated. One way to avoid this problem is by using quadratic programming formulation to minimize the deviation from the current vitamin D level of each food group. One of the main limitations of this modeling approach is that it does not encode or capture the seasonal variation of vitamin D status due to sunlight radiation and vitamin D content in food. Additionally, the effects of differences in vitamin D2 and D3 as fortification are not included in the model.

## 4. Conclusions and Future Outlook

Mixed-integer programming is shown to successfully chart a fortification strategy of vitamin D while keeping the current diet unchanged. Around 70% of the total population is shown to be lifted above the average vitamin D requirements without exceeding the tolerable upper intake level. The model is shown to be robust in that it caters to the constraints of all age groups. Mixed-integer programming can be a useful tool for policymakers to reduce deficiency of vitamin D in a population. The method also provides flexibility to encode a few choices, such as the preference for some food groups over others. The complexity introduced in the optimization model compared with the simplex model (linear programming) allows for flexibility in choosing more food groups for fortification, making it a more realistic fortification strategy.

## Figures and Tables

**Table 1:** Constraints of vitamin D consumption for different age groups of the Danish population.

Age Range	4–10 Years	11–17 Years	18–69 Years	>75 Years
Average requirement (AR) (µg/d)	7.5	7.5	7.5	7.5
Recommended intake (RI) (µg/d)	10	10	10	20
Tolerable upper level (UL) (µg/d)	50	100	100	100

AR: average requirement; RI: recommended intake; UL: tolerable upper level.

**Table 2:** Food group description considered for modeling of vitamin D fortification. Each food group has a different number of food items.

Food Group ID	Description
1	Milk and milk products
2	Cheese and cheese products
3	Ice cream, fruit ice, and other edible ices
4	Cereals and cereal products
5	Vegetables and vegetable products
6	Fruit and fruit products
7	Meat and meat products
8	Fish and fish products
9	Poultry and poultry products
10	Eggs and egg products
11	Fats, oils, and their products
12	Sugar, honey, and their products
13	Beverages
14	Spices and other ingredients
17	Other foods
25	Potatoes and their products
26	Juice

**Table 3:** Concentration composition chart. Vitamin D concentration for each food group is aggregated over all food items in the food group (without fortification) [14].

Food Group ID	Average Intake (g/Day)	Vitamin D Content (µg/g)
1	27.75	0.00056
2	5.84	0.00233
3	17.42	0.0012
4	8.92	0.00041
5	7.73	0.0
6	13.73	0.0
7	7.69	0.00538
8	6.03	0.07719
9	11.13	0.00188
10	13.03	0.04704
11	3.95	0.00506
12	5.84	0.0
13	262.9	0.0
14	3.34	0.0
17	8.66	0.0
25	59.96	0.0
26	54.63	0.0

**Table 4:** Model parameters of the optimization model. The food group chosen is a binary variable (0,1). This variable, if 1, signifies that the food group has been considered in the optimization model, and the corresponding fortification values are reported along with it.

Food Group ID	Model 1		Model 2		Model 3		Model 4	
	Food Group Chosen	Fortification (µg/g)	Food Group Chosen	Fortification (µg/g)	Food Group Chosen	Fortification (µg/g)	Food Group Chosen	Fortification (µg/g)
1	0	0	0	0	0	0	0	0
2	0	0	0	0	1	0.202	0	0
3	1	0.201	1	0.1	1	0.201	0	0
4	0	0	0	0	0	0	0	0
5	0	0	1	0.1	0	0	0	0
6	0	0	1	0.1	0	0	0	0
7	0	0	0	0	0	0	0	0
8	1	0.278	1	0.107	0	0	0	0
9	1	0.202	0	0	1	0.202	1	1.002
10	1	0.198	0	0	1	0.205	0	0
11	0	0	1	0.1	0	0	0	0
12	0	0	0	0	0	0	0	0
13	0	0	0	0	0	0	0	0
14	0	0	0	0	0	0	1	0.572
17	0	0	0	0	0	0	0	0
25	0	0	1	0.1	0	0	0	0
26	0	0	0	0	0	0	0	0

Model 1: Parameters of the mathematical optimization obtained on the entire population. Model 2: Parameters of the mathematical optimization obtained on the subpopulation of age group 11–17 years. Model 3: Parameters of the mathematical optimization obtained on the subpopulation of age group 18–69. Model 4: Parameters of mathematical optimization obtained on the subpopulation of age group 4–10 years.

**Table 5:** Comparison of the vitamin D exposure levels between the current scenario and the new scenario (after optimal fortification). Three thresholds (AR, RI, and UL) are used as references, and the number of people below the AR and RI and above the UL are reported for both scenarios, along with the total sample size for that age group. Mean and median estimated daily vitamin D intake is reported in µg/day.

Scenarios	<AR (µg/Day)	<RI (µg/Day)	>UL (µg/Day)	Mean (µg/Day)	Median (µg/Day)	Sample Size
Model 1 (Age group 4 years−75 years)						
Current	3289	3558	1	4.84	3.45	3952
New	550	908	128	19.95	17.26	
Model 4 (Age group 4 years−10 years)						
Current New	480 126	486 156	0 73	3.07 26.7	2.44 19.94	499
Model 2 (Age group 11 years−17 years)						
Current	404	417	0	3.1	2.38	431
New	1	3	5	40.8	37.3	
Model 3 (Age group 18 years −69 years)						
Current	2213	2437	0	5.3	3.8	2750
New	178	369	2	20.8	18.7	
AR: average requirement; RI: recommended intake; UL: tolerable upper level						

Model 1: Parameters of the mathematical optimization obtained on the entire population. Model 2: Parameters of the mathematical optimization obtained on the subpopulation of age group 11–17 years. Model 3: Parameters of the mathematical optimization obtained on the subpopulation of age group 18–69. Model 4: Parameters of mathematical optimization obtained on the subpopulation of age group 4–10 years.

**Table 6:** Number of persons with an intake above the AR and RI and below the UL at both scenarios (without and with fortification).

Model 1 Outcome	>AR (µg/Day)	>RI (µg/Day)	<UL (µg/Day)	Sample Size
Age group 4–10 years				
Current	19	13	499	499
Fortified	382	309	498	
Age group 11–17 years				
Current Fortified	27 321	14 265	431 431	431
Age group 18–69 years				
Current	537	313	2750	2750
Fortified	2440	2231	2747	
Age group 70–75 years				
Current	74	5	266	266
Fortified	253	155	266	

## Data Availability

The data presented in this study are available on request from the corresponding author. The data are not publicly available due to restriction of use.
